# Metabolomic Studies in Inner Ear Pathologies

**DOI:** 10.3390/metabo12030214

**Published:** 2022-02-26

**Authors:** Luc Boullaud, Hélène Blasco, Thuy-Trân Trinh, David Bakhos

**Affiliations:** 1 ENT Department and Cervico-Facial Surgery, University Center Hospital of Tours, 2 Boulevard Tonnellé, 37044 Tours, France; thuytran.trinh@gmail.com (T.-T.T.); david.bakhos@univ-tours.fr (D.B.); 2 INSERM U1253, iBrain, University of Tours, 10 Boulevard Tonnellé, 37000 Tours, France; helene.blasco@univ-tours.fr; 3 Faculty of Medicine, University of Tours, 10 Boulevard Tonnellé, 37000 Tours, France; 4 Department of Biochemistry and Molecular Biology, University Center Hospital of Tours, 2 Boulevard Tonnellé, 37044 Tours, France; 5 House Institute Foundation, Los Angeles, CA 90057, USA

**Keywords:** metabolomic, sensorineural hearing loss, perilymph

## Abstract

Sensorineural hearing loss is the most common sensory deficit. The etiologies of sensorineural hearing loss have been described and can be congenital or acquired. For congenital non-syndromic hearing loss, mutations that are related to sites of cochlear damage have been discovered (e.g., connexin proteins, mitochondrial genes, etc.). For cytomegalovirus infection or auditory neuropathies, mechanisms are also well known and well researched. Although the etiologies of sensorineural hearing loss may be evident for some patients, the damaged sites and pathological mechanisms remain unclear for patients with progressive post-lingual hearing loss. Metabolomics is an emerging technique in which all metabolites present in a sample at a given time are analyzed, reflecting a physiological state. The objective of this study was to review the literature on the use of metabolomics in hearing loss. The findings of this review suggest that metabolomic studies may help to develop objective tests for diagnosis and personalized treatment.

## 1. Introduction

Sensorineural hearing loss (SNHL) is the most common sensory deficit and a major public health problem [1]. It is estimated that hearing loss affects approximately 466 million people worldwide, or 5% of the world’s population, and 34 million of these are children. Estimates show that by 2050, 900 million people will have disabling hearing loss, representing one-tenth of the world’s population (https://www.who.int/, accessed on 8 July 2021). SNHL is a chronic disease that can affect oral language development [2], education, and social interaction. It impairs one’s quality of life by reducing social and professional relationships and communication.

SNHL may result from damage between the cochlea and the auditory cortex. It can be attributed to endocochlear lesions, neural impairment (e.g., neuropathy or vestibular schwannoma), or central hearing loss. Endocochlear damage is thought to be the leading cause of SNHL.

SNHL can be congenital (present at birth) or acquired. Congenital SNHL may be due to hereditary or non-hereditary causes. Genetic SNHL (approximately 33% of cases) is classically divided into syndromic forms, such as Waardenburg’s syndrome and Jervell and Lange-Nielsen syndrome, and non-syndromic forms, e.g., autosomal dominant, autosomal recessive, X-linked, and mitochondrial. Genetic analysis has allowed for a better understanding of the pathophysiological mechanisms of SNHL. For example, some genes can affect inner ear homeostasis (e.g., mutation in GJB2 coding for connexin 26) or mechano-electrical transduction (e.g., mutation in potassium voltage-gated channel subfamily Q member 4 (KCNQ4) coding for potassium channels). Non-hereditary causes include prenatal infections (e.g., cytomegalovirus infection), prematurity, anoxia, preeclampsia, maternal diabetes, and drug or alcohol use during pregnancy.

The major causes of acquired SNHL are aging, ototoxic drugs, genetic mutations, sudden hearing loss, and external factors (e.g., smoking) [2,3]. However, in approximately 50% of post-lingual cases of SNHL, the etiology remains unknown despite clinical, audiometric, radiological, or genetic findings [4]. For these patients, the site of the lesion and the molecular mechanisms involved remain unclear. SNHL may be related to the involvement of approximately 30 different cell types in the cochlea and to the alteration of the auditory nerve.

Five main pathways have been described for the cellular mechanisms involved in SNHL [2,3]: (1) The accumulation of reactive oxygen species (ROS) and reactive nitrogen species (RNS) in the cochlea that contribute to oxidative stress, resulting in apoptosis via a cascade of reactions. (2) An increase in free Ca^2+^ in hair cells, resulting in mitochondrial perturbation, apoptosis, and hair cell death. (3) The involvement of mitogen-activated protein kinase (MAPK), which is associated with signaling proteins that help to activate stress-activated protein kinase, a key mediator of oxidative stress and inflammation related to cell death. (4) Programmed hair cell death via the intracellular or extracellular pathway (caspase). (5) Infection and/or inflammation that induces apoptosis via an innate immunity-based response, promoting pathogen death. While many pathways leading to ciliated cell loss and nerve cell loss have been described and appear to be interrelated, there are currently no available diagnostic tools with which to characterize the site of injury in the cochlea and the intracellular mechanisms of SNHL [5].

The cellular mechanisms involved in hearing loss can be intricately linked. Currently, there is no tool available with which to characterize the pathophysiological mechanisms in SNHL of an undetermined etiology, or to identify the lesion sites involved. This makes an accurate etiological diagnosis challenging, as no specific biomarker has been identified with which to characterize SNHL in terms of its origin, severity, and evolution. Identifying biomarkers linked to the metabolic mechanisms involved could allow for better characterization of SNHL, better prediction of the evolution of SNHL, and the personalized management of SNHL. Modifications to metabolic pathways can be investigated using metabolomic analysis, an emerging technique that can be used to identify metabolic biomarkers via the analysis of individual metabolites present in an organic sample. The “metabolome” refers to all metabolites of biological origin below 1500 daltons (i.e., a low molecular weight) [6], and provides insight into physiological or pathological mechanisms at a given time. A specific feature of the metabolome is that it is dynamic and subject to the influence of environmental factors, such as diet, toxins, and disease [7]. Metabolomic studies use several analytical techniques, including nuclear magnetic resonance spectrometry (NMR), gas chromatography-mass spectrometry (GC-MS), and liquid chromatography-mass spectrometry (LC-MS) [8,9]. Such research will help in the development of therapeutics according to identified biomarkers. For example, metabolomic analysis has been used to identify some mutations and to better understand the pathophysiological aspects of hearing, balance, and vision loss for patients with Usher syndrome [10,11]. This research has allowed for the investigation of gene therapy to restore auditory, balance, and vision functions.

Metabolomic research appears to be a promising tool with which to study SNHL. Metabolomic analysis may provide additional data regarding the known etiologies of deafness and help to better categorize SNHL when etiology remains unknown. The objectives of this review are to alert readers to the growing interest in metabolomic approaches for the study of inner ear pathologies, to provide innovative scientific findings from metabolomics studies, and to discuss new experimental designs with which to better understand progressive hearing loss.

## 2. Methods

This systematic review was written in accordance with the Cochrane Handbook for Systematic Reviews of Interventions Version 5.2.0 (June 2017) and followed the PRISMA (Preferred Reporting Items for Systematic Review and Meta-Analysis recommendations) method [12]. The PROSPERO database was searched and no completed or ongoing studies on this topic were identified.

### 2.1. Research Strategy

This systematic literature review was conducted by searching medical literature analysis and retrieval system online (MEDLINE; PubMed interface), Excerpta Medical dataBASE (EMBASE; Elsevier interface), Cochrane Central Register of Controlled Trials (CENTRAL; Wiley interface), International Standard Randomised Controlled Trial Number (ISRCTN) registry (http://www.isrctn.com; accessed on 12 September 2021), https://www.clinicaltrials.gov/ (accessed on 12 September 2021), and Scientific Electronic Library Online (SciELO). The search began in November 2020 and was completed in August 2021.

The medical subject headings (MeSH) thesaurus was used to search the MEDLINE database. The search terms used were “Hearing loss” and “Metabolomic”, “Deafness” and “Metabolomic”, and “Metabolomic” and “Inner Ear”. The Cochrane Central Register of Controlled Trials (CENTRAL) was searched for “Hearing loss” and “Metabolomic”, “Deafness” and “Metabolomic”, and “Metabolomic” and “Inner Ear”.

### 2.2. Selection Criteria

The study selection was carried out according to the “Participants, Interventions, Comparisons, Outcomes” (PICO) characteristics. Biomarker studies in patients with SNHL or animal models of deafness based on a metabolomic approach were included. The eligibility criteria were English language studies dealing with metabolomic analysis related to hearing loss, regardless of the fluid that was analyzed. All articles without metabolomic analysis or without the analysis of hearing loss were excluded.

### 2.3. Data Extraction

Two authors independently collected papers based on titles, abstracts, and keywords. The research was conducted according to the exclusion and eligibility criteria defined above. The data extraction method was blinded and double-reviewed. In the case of disagreement, the two authors met to reach a consensus, and the final adjudication was based on the expert opinion of the senior author.

Here, the methodologies of the studies are described, focusing on the analytical technique used, the types of samples studied, and the types of experiments performed. The main results concerning the metabolites found and the metabolic pathways are also presented.

## 3. Results

**Figure 1:**
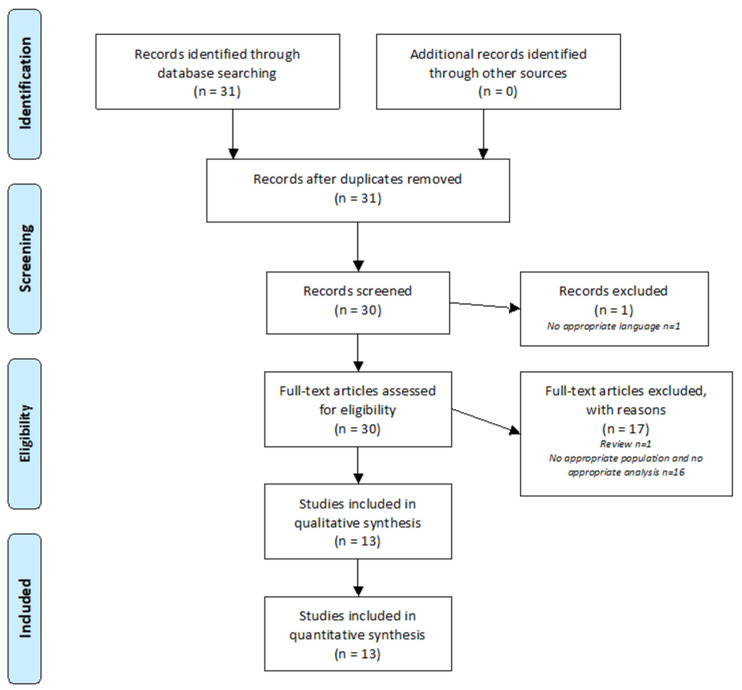
PRISMA Flow Diagram. The PRISMA diagram details the search and selection process applied during the review.

Figure 1 summarizes the analysis of the 31 articles identified using the PRISMA method. One article was excluded because it was written in Chinese. Thirty articles were analyzed. After review, 17 articles were excluded because they did not address SNHL and/or metabolomic analysis. A total of 13 articles were included in this review.

### 3.1. Types of Samples

In the included studies, different types of samples were subjected to metabolomic analysis. Perilymph was used in six of thirteen studies; perilymph has the advantage of being closer to metabolic mechanisms because hair cell bodies are immersed in this fluid [13,14] The volumes obtained for analysis were small, ranging from <1 μL in humans [8,15] to 5 μL in guinea pigs [16,17]. Two studies collected human perilymph during cochlear implantation [8,15]. Three studies involved the metabolomic analysis of guinea pig perilymph [16,17,18]. One study [19] analyzed mouse temporal bone containing endocochlear fluids and bone constituents.

Blood plasma was collected in three studies to investigate cisplatin-induced changes at the systemic level [20], the metabolomic blood profile in a patient with hearing loss due to mitochondrial mutation [21], and changes in the metabolomic analysis of plasma following noise exposure in humans [22]. The volumes used for analysis were larger but required collection in a heparinized tube followed by centrifugation before freezing [20,21,22].

Urine, composed of the final products from metabolic breakdown, was analyzed in two studies [7,23]. The volume used was 1 mL, collected from first morning urine [7,23].

In another study, rat brain tissue was subjected to metabolomic analysis to better understand metabolic changes at the cortical level in the genesis of tinnitus induced by acoustic trauma [24].

One study investigated dexamethasone-induced changes in a ciliated cell-like HEI-OC1 cell line [25]. This was a model for an in vitro analysis of drug metabolic phenomena in cells.

Different types of samples are associated with different functions and purposes. Perilymph was the most commonly used fluid in the studies presented, given the hypothesis that some metabolomic changes could be observed in perilymph following induced SNHL. Perilymph is located in the inner ear, close to pathophysiological events; however, its volume is small, and collection requires invasive sampling. Perilymph samples may be contaminated, and volumes may be insufficient [18]. For these reasons, analytical techniques must be validated. In addition, routine collection from a patient may result in deafness and significant surgery-related morbidity. Urine and blood are more readily available with less morbidity. If a specific biomarker of an etiology is found, it may allow us to make an etiological diagnosis and to propose personalized treatment in routine clinical practice.

### 3.2. Analytical Techniques

The analytical methods used to perform the metabolomic analyses were NMR spectrometry in one study [7], GCMS in four studies [16,23,24,25], and LCMS in eight studies [15,17,18,19,20,21,22]. Mass spectrometry (GCMS and LCMS) has a greater sensitivity than NMR spectrometry; however, this technique suffers from limited reproducibility [26]. LCMS seems to be more appropriate to explore low volumes of samples [8] and allows for the detection of thermally labile and highly polar compounds [17]. Table 1 shows the selected metabolomics studies of SHNL.

LCMS is the most commonly used approach in different studies. No consensus exists on the analytical method that is most appropriate in this context; the choice depends on the volume of the sample inherent to its nature and the targeted metabolic pattern. Consequently, the exploration of the perilymph by LCMS seems to be the most informative strategy with which to analyze the pathophysiology of hearing loss

### 3.3. Animal Studies

Four studies investigated metabolomic changes in animals following noise exposure.

#### 3.3.1. Mice

One study performed a metabolomic analysis of the entire cochlea located in the temporal bone of mice after noise-induced trauma [23]. The aim was to examine the metabolic changes in the inner ear after noise exposure. A noise level (8–16 kHz) of 100 dB for 2 h was used. The authors identified 220 metabolites, 40 of which appeared to be involved in the pathophysiological mechanisms of noise-induced SNHL. Twenty-five of these genes were increased, belonging to the nucleotide family, cofactors, carbohydrates, alanine, aspartate, purine, glutamine, and glutamate. Fifteen metabolites were decreased (amino acids, such as arginine, methionine, phenylalanine, tyrosine, and tryptophan). The metabolic pathways involved were alanine/aspartate/tryptophan, purine metabolism, and phenylalanine/tyrosine/tryptophan biosynthesis. This study showed a reliable methodology with promising results. The results contribute to knowledge regarding the pathophysiological mechanisms of noise-induced trauma, showing the involvement of oxidative stress, which contributes to the understanding of the complex neurotransmission and modulation pathways. The results also suggest an important role for glutamate in the pathophysiology of SNHL due to synaptopathy following acoustic trauma.

#### 3.3.2. Rats

In one study, metabolic brain changes were investigated in rats following sound exposure with a sound level of 110 dB at 16 kHz for 1 h [24]. Auditory thresholds were measured after acoustic shock using auditory brain responses, and the presence of tinnitus was observed using behavioral studies. Eighty-eight metabolites were identified, 17 of which were involved in sound trauma. The four associated metabolic pathways were the glutathione pathway, the aspartate/alanine/glutathione pathway, the arginine/proline/glycine pathway, and the serine/threonine pathway [24]. The authors suggested that glutathione and urea metabolism appeared to play a role in sound trauma-induced hearing loss. Glutathione is an antioxidant in cells, and urea metabolism may play an important role in the maintenance of neuronal function.

#### 3.3.3. Guinea Pigs

Two studies focused on noise-induced changes in the metabolomic analysis of perilymph [16,17]. One study performed a metabolomic analysis of guinea pig perilymph after noise exposure (126 dB at 4 kHz for 2 h) [16]. Seventy-seven metabolites were identified in the perilymph fluid. The levels of 10 metabolites (3-hydroxy-butyrate, glycerol, fumaric acid, galactosamine, pyruvate, oxaloacetic acid, phosphate, meso-erythritol, citric acid, isocitric acid, mannose, and inositol) were altered following noise exposure. Another study investigated the metabolomic profile of perilymph after noise-induced hearing loss using an impulse noise (400 pulses at 156 dB for 4 min) with or without H_2_ gas [17]. Ten guinea pigs were exposed to noise alone, 10 to noise and H_2_ gas, four to H_2_ gas alone, and two were not exposed. The results showed an increase in eight metabolites (including pantothenic acid, creatine, butyrylcarnitine, and short-chain acyl-carnitine) and a decrease in stachydrine and homostachydrine in the noise-exposed group, compared to the H_2_-exposed group. Stachydrine and homostachydrine decreased in the noise-exposed group alone, with similar levels observed in the noise-exposed with H_2_ gas, H_2_ gas alone, and unexposed groups. Acyl-carnitines were increased in the noise-exposed group compared to the noise-exposed group with H_2_ gas, and were involved in oxidative stress. Audiometric results were significantly improved in guinea pigs exposed to noise combined with H_2_ gas compared to noise alone. These two articles showed metabolic differences following noise exposure in guinea pig perilymph; however, the differences did not allow for any conclusions.

Metabolomic analysis following cisplatin-induced ototoxicity has been the subject of two studies [18,20]. One article investigated the protective effect of H_2_ gas inhalation on cisplatin-induced ototoxicity in 34 guinea pigs via a morphological, immunohistochemical, and metabolomic analysis of the perilymph [18]. The authors identified 50 metabolites. Exposure to cisplatin resulted in changes in 11 metabolites, including choline, creatine, methylguanine, carnitine, proline, cytosine, ecgonine, hydroxystrachydine, homostrachydine, creatinine, and dimethylarginine. H_2_ gas inhalation showed an otoprotective effect against cisplatin-induced toxicity at the auditory level, as well as immunohistochemically. Subsequently, another study examined metabolomic changes in guinea pig blood serum after an intravenous injection of cisplatin or NaCl [20]. Four significant metabolite changes were found for N-acetylneuraminic acid, l-acetylcarnitine, ceramide, and cysteinylserine.

### 3.4. Human Biomarker Research

The literature review found five out of thirteen articles on metabolomic analysis and SNHL in humans.

#### 3.4.1. Urine Sample

One study performed a metabolomic analysis of urine to look for a correlation between presbycusis and renal dysfunction as defined by traditional Chinese medicine. The authors found differences in 23 metabolites between subjects with presbycusis and those with normal hearing. After analysis by the Kyoto Encyclopedia of Genes and Genomes (KEGG) software, these metabolites were found to be involved in the metabolism of glutathione, amino acids, glucose, N-methyl-D-aspartate (NMDA), and gamma-aminobutyric acid (GABA) receptors [19]. Another study performed a metabolomic analysis of urine from patients with sudden hearing loss [7] and included 26 subjects (21 patients with sudden hearing loss and five normal-hearing controls). Of the 21 patients with sudden hearing loss, eight recovered their hearing thresholds after dexamethasone treatment, while 13 had no improvement in hearing performance. Metabolic analysis showed an increase in ß-alanine, 3-hydroxybutyrate, and trimethylamine N-oxide (TMAO) in the non-responders. Responders to the treatment showed an increase in citrate and creatine.

The identification of specific biomarkers of hearing impairment in urine may help to better understand the pathophysiological mechanisms and to classify the etiologies of sudden or mitochondrial deafness.

#### 3.4.2. Perilymph Sample

One article performed a feasibility study for the metabolomic analysis of a small amount of human perilymph (<1 μL) collected during cochlear implantation using LCMS [8]. Ninety-eight metabolites were identified in the perilymph of subjects with SNHL. The most frequent metabolites were asparagine and lactate [8]. An analysis between the metabolomic profile of the perilymph and the duration of hearing loss was performed [15]. A total of 106 different metabolites were found using the LCMS technique in the 19 included patients [8]. A significant correlation was observed between N-acetylneuraminate and the duration of hearing loss. This metabolite is derived from sialic acid, which is usually located on the terminal part of glycoproteins and glycolipids on the cell membrane surface. N-acetylneuraminate is also a degradation product of glucose.

#### 3.4.3. Plasma Sample

One study performed an exome and plasma metabolome analysis of a child with mitochondrial kidney disease (RMND1 mutation) and deafness. Ceramide accumulation was observed, which provided a better understanding of the pathophysiological mechanism involved [21]. Another study analyzed the metabolomic profile of plasma in noise-exposed patients [22]. The analysis was performed by LCMS in 62 patients and 62 controls. The analysis identified 207 metabolites. Twenty significantly different metabolites were found: 12 metabolites were increased and eight were decreased. They were involved in seven different metabolic pathways (glycerophospolipids, glycerophosphatidylinositol, choline, autophagy, alpha-linoleic acid, linoleic acid, and endocannabinoids).

#### 3.4.4. In Vitro Cells

One study investigated dexamethasone-induced changes in a ciliated cell-like cell line at the metabolic level under two growth conditions [25]. They were compared in four groups using GCMS. The cells were House Ear Institute-Organ of Corti 1 (HEI-OC1) cells, which have the ability to differentiate into hair cells under certain conditions. Two groups were composed of differentiating cells: one group was exposed to dexamethasone, and the other was a control group. The other two groups consisted of differentiated cells: one exposed to dexamethasone and the other being the control group. Seventy metabolites were identified. There was little variation in the metabolic profile of the differentiating cells, whereas the profile was variable for the differentiated cells. Dexamethasone increased the abundance of energy metabolites, leading to the increased availability of glucose and substrates for glycolysis, increased production of ATP by beta-oxidation, and decreased consumption of amino acids. A role of the pantothenate and CoA pathways at the metabolic level induced by dexamethasone was suggested.

Of the seven papers, two investigated perilymph, two urine, two serum, and one HEI-OC1 cells in vitro. With the exception of two papers [21,22], the etiologies were unknown. This allowed for the observation of the impairment of certain metabolic pathways and correlations, such as with N-acetylneuraminate and the duration of hearing loss [15] or ceramide accumulation [21]. Metabolomic analysis using in vitro models may allow for preliminary discovery of the pathophysiology of hearing loss and may be an initial step towards using drug therapeutics in animals. Given the easy access, using blood serum or urine may allow for the easy clinical diagnosis of etiology. It is necessary to define beforehand the metabolomic profiles of each etiology of deafness via the identification of specific biomarkers. The results are encouraging, but the disparity between the fluids and the etiologies studied does not allow conclusions to be drawn, due to the limited number of studies regarding metabolomics and hearing loss. It is important to continue this research to expand our current knowledge of the pathophysiological mechanisms involved in SNHL. Metabolomics opens many doors to improve pathophysiological knowledge, as a diagnostic tool available to clinicians or to achieve personalized and curative treatment of hearing loss.

## 4. Discussion

### 4.1. Slow Progression of SNHL-Related Metabolomics Studies

Most of the included metabolomic studies investigated noise-induced hearing loss [16,17,19,22,24]. Metabolic changes were observed for pathways involving glutathione, arginine/proline/glycine, alanine/aspartate/glutathione, purine, and phenylalanine/tyrosine/tryptophan, as well as the modification of some metabolites (increase in 3-hydroxy-butyrate). The fluids investigated in these studies were the perilymph [16,17] plasma [22], temporal bone [19], and brain tissue [24]. The patterns of noise-induced hearing loss varied between studies. It is difficult to draw conclusions given the small number of studies and differences in study designs; however, the results are encouraging.

Metabolomic changes following cisplatin-induced ototoxicity were investigated in two studies on guinea pigs using perilymph analyzed by LCMS with the addition of H_2_ gas [18] and blood analyzed by LCMS [20]. Changes were observed for N-acetylneuraminic acid, L-acetylcarnitine, ceramide, cysteinylserine, choline, creatine, methylguanine, carnitine, proline, cytosine, ecgonine, creatinine, and dimethylarginine. The fluids analyzed were different between the two studies, as well as the objective; this does not allow any conclusions regarding the involvement of a metabolic pathway following cisplatin exposure.

In another study, sudden hearing loss was explored by the metabolomic analysis of urine using NMR spectrometry [7]. The results showed some differences in metabolites between good and poor responders following corticosteroid treatment (ß-alanine, 3-hydroxybutyrate, and trimethyl N-oxide (TMAO)). While these initial results are interesting, further studies are needed of urinary metabolites as biomarkers of treatment response in patients with sudden hearing loss.

In two studies with humans, the metabolomic analysis of perilymph was performed by LCMS for cochlear implant candidates and the perilymph was sampled during surgery [8,15]. Too few studies have been conducted thus far in humans. Systematic sampling for various known etiologies would allow for the identification of different metabolomic profiles related to these etiologies. Such sampling is only possible in patients with hearing impairment during cochlear implantation.

Another research axis using metabolomic analysis could be Ménière’s disease. Previous studies have identified endolymphatic hydrops in these patients. However, the pathophysiological mechanisms are not yet fully understood, although genetic, vascular, immunological origin, the aberrant expression of aquaporins, or a combination of causes have been proposed [27]. Several research teams have been interested in identifying biomarkers and mechanisms in this pathology. Using miRNA profiles, some authors found altered perilymph linked with Ménière’s disease, which could serve as a potential biomarker [27]. Proteonomic analysis has allowed for the identification of protein short-chain dehydrogenase/reductase family 9C member 7 in perilymph from patients with Ménière’s disease [28]. Metabolomic analysis may also aid in the identification of biomarkers in these patients.

Metabolomic analysis is an innovative and recent technique. The analysis approach depends on the type and volume of the sample, and the type of metabolites sought. Due to inter-individual variability and the role of environmental conditions [29], it is necessary to control these parameters and repeat measurements to investigate the most promising metabolic pathways. Metabolomic analysis is positioned to elucidate the pathophysiology of SNHL and may allow for better diagnosis and personalized curative treatment.

### 4.2. What Is the Most Promising Biological Fluid for Studies of SNHL?

Among the different types of samples analyzed in the included studies, perilymph is likely to be in the closest proximity to pathophysiological events; hair cell bodies are bathed in perilymph. Metabolomic analysis has shown a difference in metabolites between blood and perilymph in the baseline state [16]. There is a difference in membrane components, which results in a physiological difference in metabolomic analysis between the different body fluids. Urine and plasma are attractive types of samples because they are easily collected [30]. The possibility of studying hair cells in vitro is also very desirable [25], allowing for the observation of metabolic relationships for a drug or for an etiology. Furthermore, in vitro hair cell studies could be used as a prerequisite before animal experimentation.

Currently, there are no comparative metabolomic studies on the different fluids that can be sampled to determine which fluid is most promising. In the future, it would be interesting to define the best sample to use in terms of ease of collection, sensitivity, and specificity in the context of SNHL.

### 4.3. Progress in Metabolomic Analysis Methods on Constrained Biological Fluids

The evolution of analytical techniques has allowed for the characterization of a larger number of metabolites. In 2015, 77 metabolites were found in perilymph with GCMS [16]; in 2019, 220 metabolites were characterized using LCMS [19]. LCMS has been validated, allowing mass spectrometry and liquid chromatography to be performed on very small samples (<1 μL) [8]. LCMS for perilymph analysis is based on two complementary chromatographic media: reversed-phase (RP C18) and hydraulic interaction liquid chromatography (HILIC). For polar/ionic molecules that are not well retained with RP C18 (e.g., amino acids and sugars), separation using HILIC is a complementary option that allows for improved results. By combining these two types of columns, 98 metabolites were detected, 25 of which were found in both RP C18 and HILIC media [8]. These results show the advantage of using several types of chromatographic columns, allowing for a wider detection of metabolites, as 43 metabolites were detected using RP C18 and 30 metabolites using HILIC [8].

### 4.4. Next Steps Needed in the Field of Metabolomic Studies

In the future, it will be necessary to conduct complementary studies in animal models to discover metabolic profiles linked with hearing loss phenotypes. For example, noise-induced hearing loss can be explored by comparing normal perilymph to perilymph following noise-induced hearing loss; differences in metabolic profiles may explain the pathophysiology.

Sampling the perilymph from a large cohort of patients might allow for the identification of biomarkers that could explain the pathophysiological mechanisms associated with SNHL or be related to the duration and etiology of SNHL. For example, studies of perilymph during cochlear implantation have provided interesting information on the etiologies of SNHL and on the duration of hearing loss, allowing for improved management of SNHL [15,28]. Further development of metabolomic analysis techniques will likely increase the currently fragmented knowledge of SNHL etiologies and allow for personalized treatment for patients with SNHL [31].

## 5. Limits

To date, there are few studies regarding metabolomic analysis and hearing loss, and even fewer on the metabolomic analysis of perilymph. Furthermore, the metabolomic analysis of perilymph in normal hearing humans is not ethically feasible due to the induction of cophosis during sampling. This could be overcome by proposing a metabolomic study of perilymph in organ donors. The studies conducted to date in humans have all been carried out on patients receiving cochlear implantation for profound deafness; however, the etiology and duration of the deafness are not always easy to define. In addition, most of the experimental studies have been performed on small animals (mice, rats, guinea pigs). It would be interesting to study the perilymph in larger mammals, such as sheep or dogs, which are physiologically and anatomically similar to humans [32,33]. For example, 72 metabolites were found in guinea pig perilymph [16], and 98-106 were found in humans [8,15], but only 15 metabolites were common to both species. A species-related metabolic difference may explain these divergent results between guinea pig and human perilymph.

## 6. Conclusions

The current methods for the exploration of deafness with subjective and objective audiometry do not allow for the precise identification of damaged sites within the cochlea for patients where the etiology of SNHL is unknown. The purpose of this review was to assist investigators and Ear, Nose and Throat (ENT) surgeons to better understand the value of metabolomic analysis for patients with SNHL. Further research is needed to define normal and pathological states using metabolomic analysis. This will help to define biomarkers linked with etiology, which has great clinical relevance. Metabolomic analysis will also increase our understanding of the pathways involved and foster the development of new therapies. One of the challenges faced in these future studies will be to sample the perilymph without adverse effects.

## Figures and Tables

**Table 1:** Studies according to publication journal, animal type, subject, pathology type, sample type, and metabolomic analysis technique.

Authors, Year	Participants	Pathology	Sample	Metabolomic Technique	Main Findings
Gaboon NEA et al., 2020 [17]	Hu	Mitochondrial disease	Blood	LCMS	Metabolomic analysis of renal disease with HL induced by a RMND1 mutation revealed ceramide accumulation
Trinh TT et al., 2019 [11]	Hu	SNHL	Perilymph	LCMS	Relationship between metabolomics profile and duration of HL
Mavel S et al., 2018 [6]	Hu	SNHL	Perilymph	LCMS	Feasibility of metabolomic analysis of human perilymph
Carta F et al., 2017 [5]	Hu	Metabolomic study of urine in sudden HL	Urine	NMR spectrometry	Metabolomic profiles differ depending on auditory recovery following dexamethasone treatment
Dong Y et al., 2013 [19]	Hu	Presbyacusis and renal dysfunction	Urine	GCMS	Difference in metabolomic profile for patients with renal failure, with and without hearing impairment
Miao L et al., 2021 [18]	Hu	NIHL	Blood	LCMS	NIHL-induced changes in the plasma profile
Pirttilä K et al., 2019 [13]	GP	NIHL	Perilymph	LCMS	Difference in terms of metabolomic profiles between NIHL and NH
Fransson A et al., 2017 [14]	GP	Ototoxicity to cisplatin	Perilymph	LCMS	Cisplatin-induced changes in the perilymph metabolomic profile
Videhult Pierre et al., 2017 [16]	GP	Ototoxicity to cisplatin	Blood	LCMS	Cisplatin-induced changes in the serum metabolome
Fujita T et al., 2015 [12]	GP	NIHL	Perilymph	GCMS	NIHL-induced changes in the perilymph metabolomic profile
Ji L et al., 2019 [15]	Mouse	NIHL	Perilymph Temporal bone	LCMS	NIHL-induced changes in the temporal bone metabolomic profile
He J et al., 2017 [20]	Rat	NIHL	Cerebral tissue	GCMS	Cerebral metabolic changes in rats after exposure to acoustic trauma
Kather M et al., 2021 [21]	Cells (in vitro model)	Research	Cell line HEI-OC1	GCMS	Dexamethasone-induced metabolic changes in a ciliated cell line under both growth conditions

Hu—human; GP—guinea pig; HL—hearing loss; NH—normal hearing; NIHL—noise-induced hearing loss; SNHL—sensorineural hearing loss; NMR—nuclear magnetic resonance; GCMS—gas chromatography mass spectrometry; LCMS—liquid chromatography mass spectrometry.
